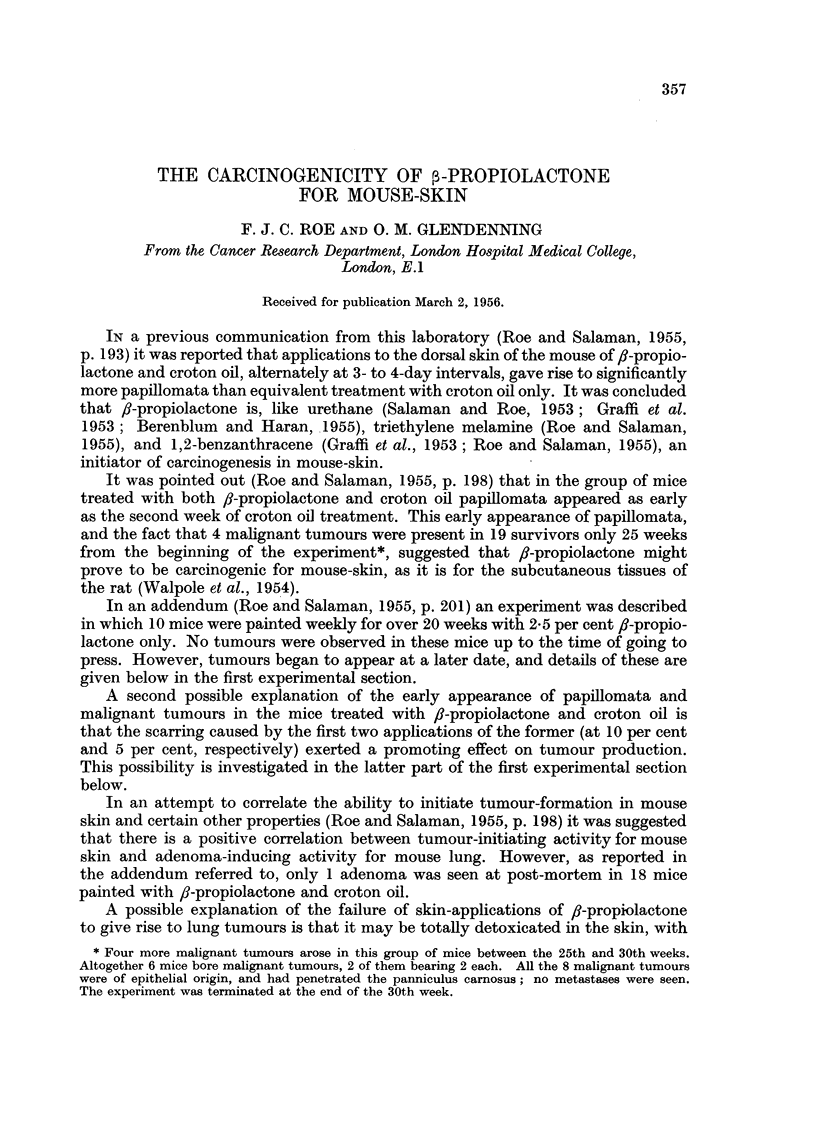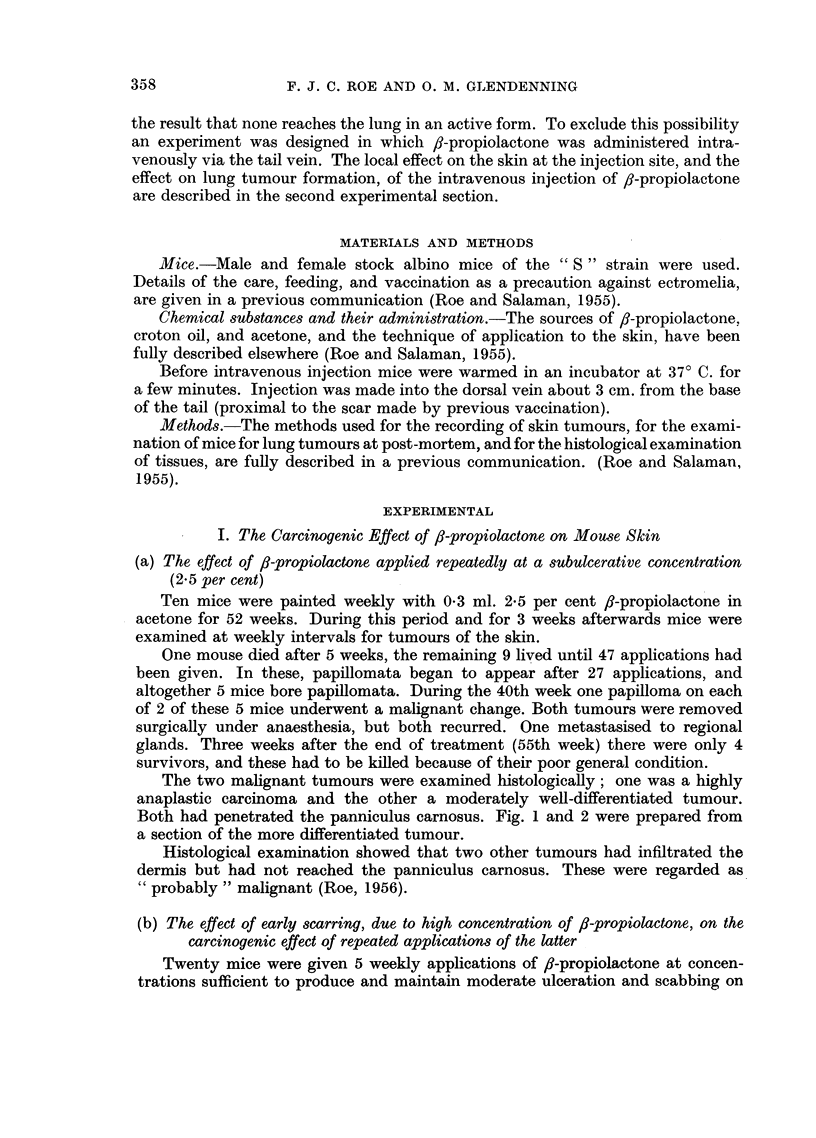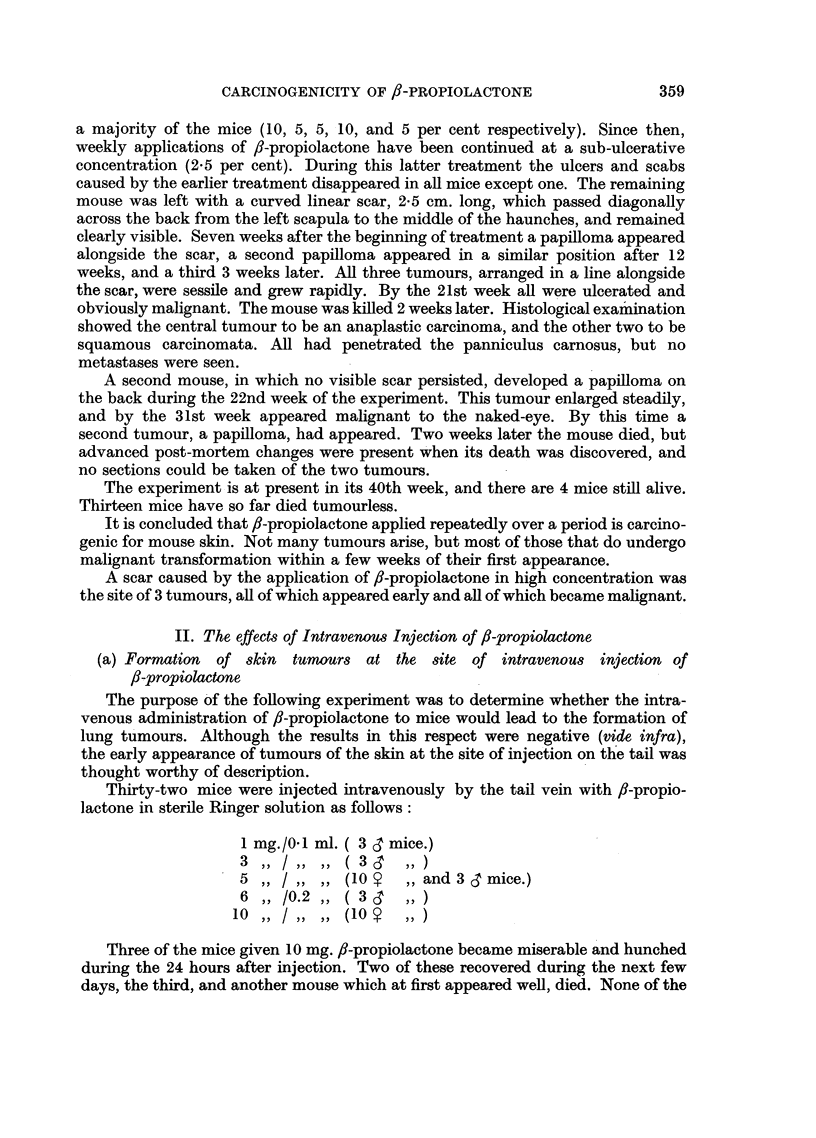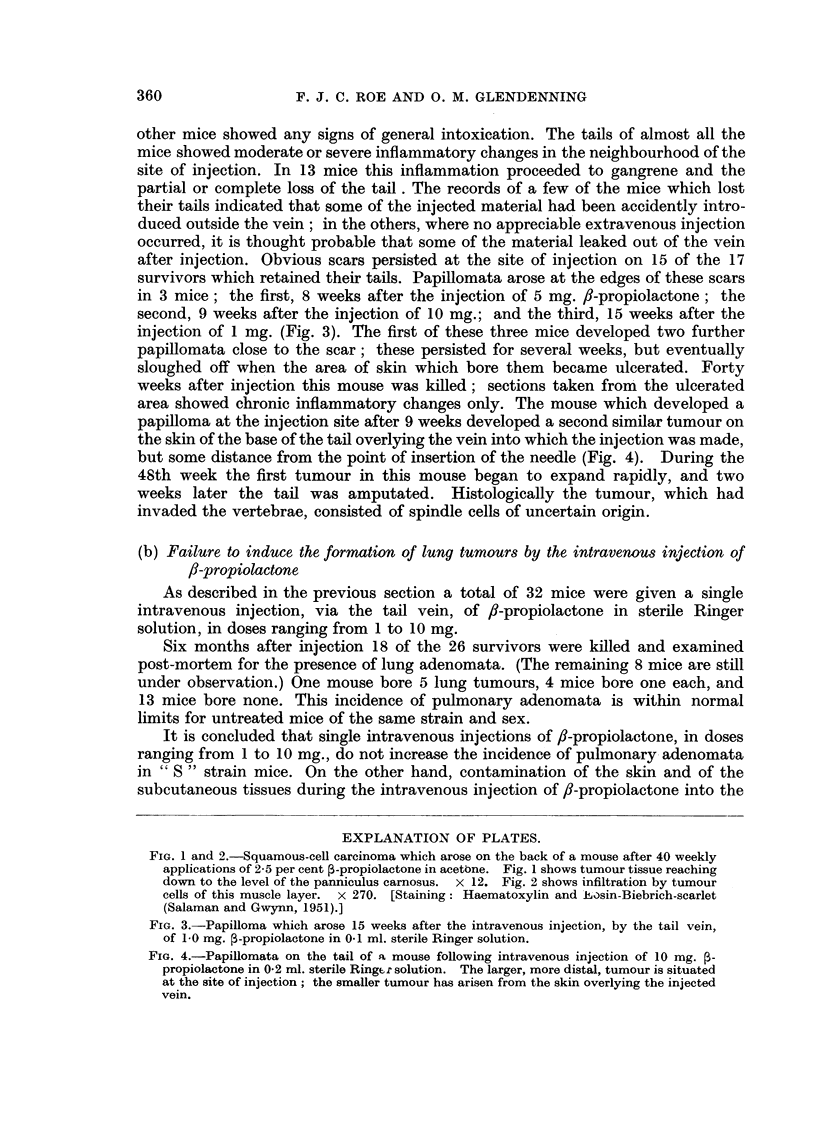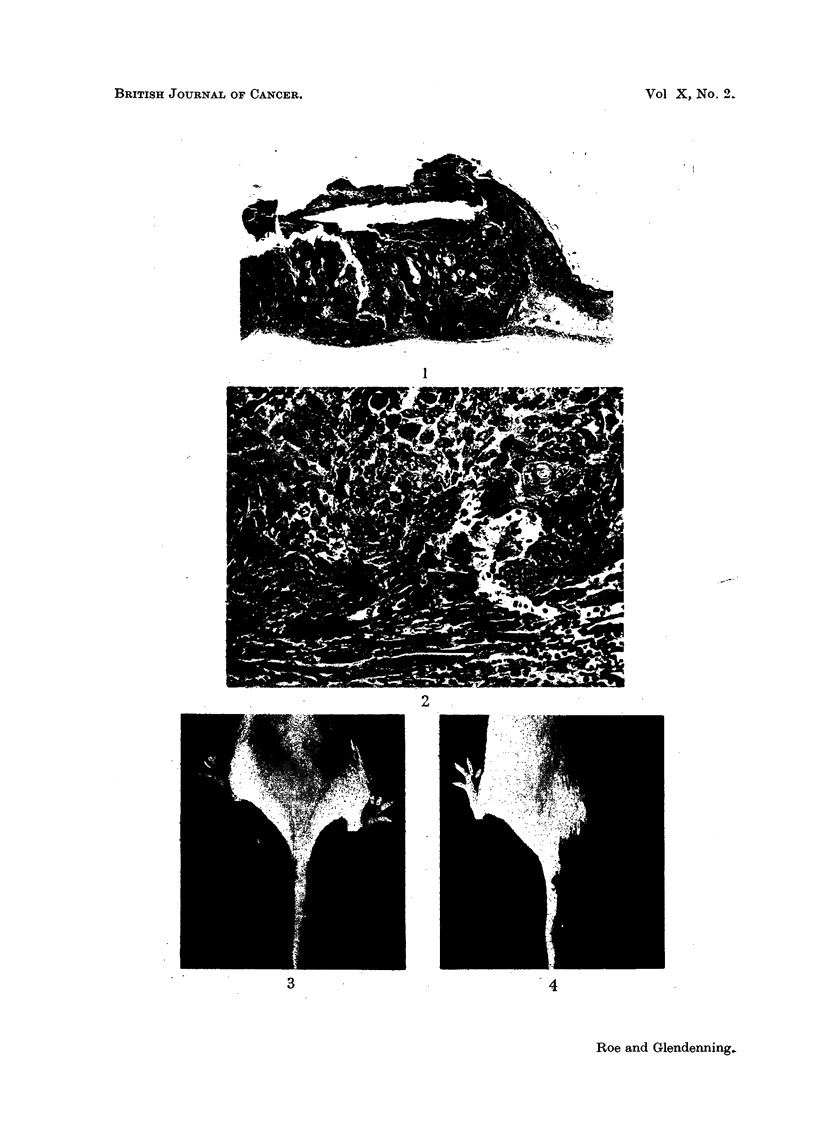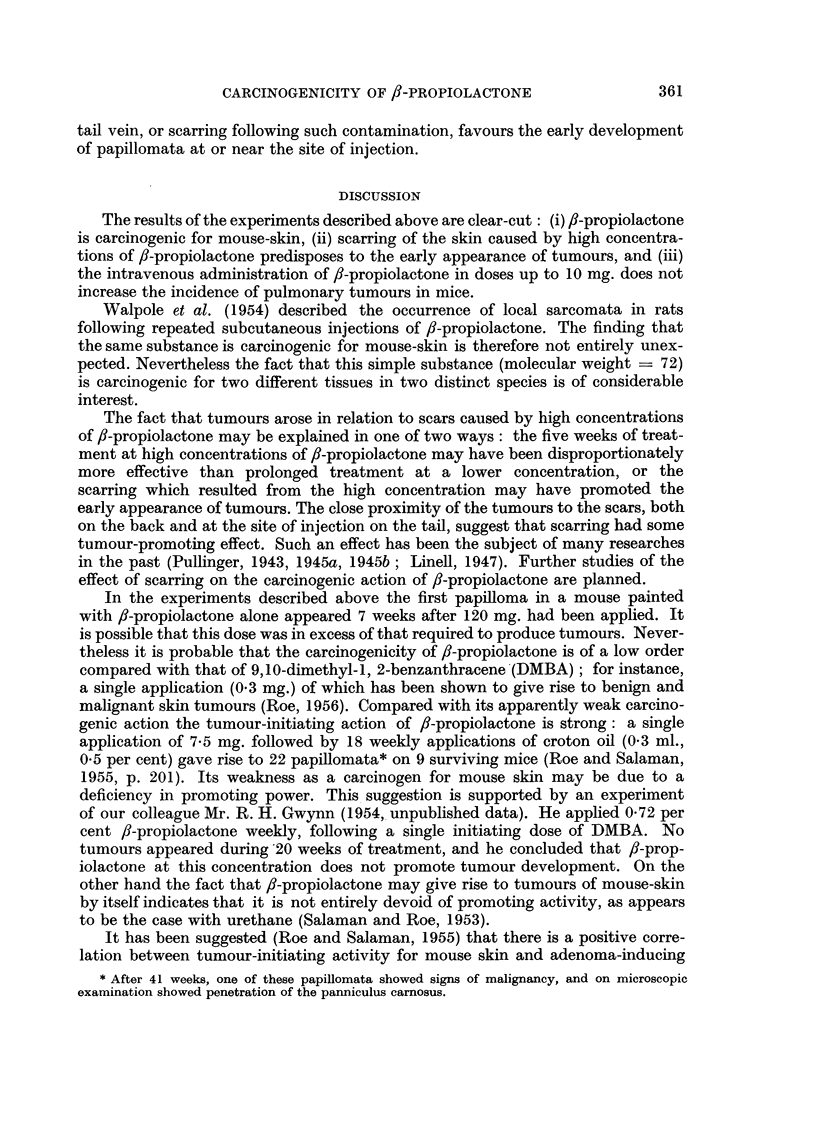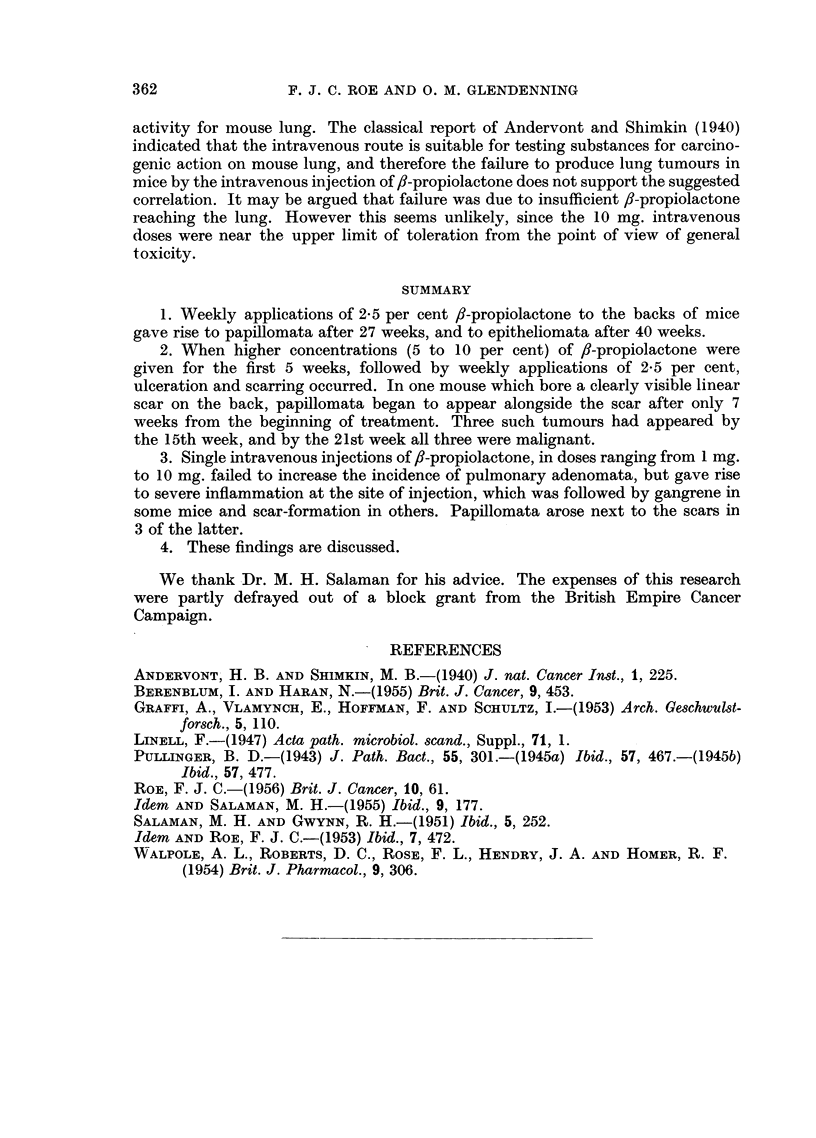# The Carcinogenicity of β-Propiolactone for Mouse-Skin

**DOI:** 10.1038/bjc.1956.41

**Published:** 1956-06

**Authors:** F. J. C. Roe, O. M. Glendenning

## Abstract

**Images:**


### THE CARCINOGENICITY OF β-PROPIOLACTONE FOR MOUSE-SKIN

F. J. C. ROE AND O. M. GLENDENNING

*From the Cancer Research Department, London Hospital Medical College, London, E.1*

Received for publication March 2, 1956.

IN a previous communication from this laboratory (Roe and Salaman, 1955, p. 193) it was reported that applications to the dorsal skin of the mouse of β-propiolactone and croton oil, alternately at 3- to 4-day intervals, gave rise to significantly more papillomata than equivalent treatment with croton oil only. It was concluded that β-propiolactone is, like urethane (Salaman and Roe, 1953 ; Graffi *et al.* 1953 ; Berenblum and Haran, 1955), triethylene melamine (Roe and Salaman, 1955), and 1,2-benzanthracene (Graffi *et al.*, 1953 ; Roe and Salaman, 1955), an initiator of carcinogenesis in mouse-skin.

It was pointed out (Roe and Salaman, 1955, p. 198) that in the group of mice treated with both β-propiolactone and croton oil papillomata appeared as early as the second week of croton oil treatment. This early appearance of papillomata, and the fact that 4 malignant tumours were present in 19 survivors only 25 weeks from the beginning of the experiment*, suggested that β-propiolactone might prove to be carcinogenic for mouse-skin, as it is for the subcutaneous tissues of the rat (Walpole *et al.*, 1954).

In an addendum (Roe and Salaman, 1955, p. 201) an experiment was described in which 10 mice were painted weekly for over 20 weeks with 2·5 per cent β-propiolactone only. No tumours were observed in these mice up to the time of going to press. However, tumours began to appear at a later date, and details of these are given below in the first experimental section.

A second possible explanation of the early appearance of papillomata and malignant tumours in the mice treated with β-propiolactone and croton oil is that the scarring caused by the first two applications of the former (at 10 per cent and 5 per cent, respectively) exerted a promoting effect on tumour production. This possibility is investigated in the latter part of the first experimental section below.

In an attempt to correlate the ability to initiate tumour-formation in mouse skin and certain other properties (Roe and Salaman, 1955, p. 198) it was suggested that there is a positive correlation between tumour-initiating activity for mouse skin and adenoma-inducing activity for mouse lung. However, as reported in the addendum referred to, only 1 adenoma was seen at post-mortem in 18 mice painted with β-propiolactone and croton oil.

A possible explanation of the failure of skin-applications of β-propiolactone to give rise to lung tumours is that it may be totally detoxicated in the skin, with

* Four more malignant tumours arose in this group of mice between the 25th and 30th weeks. Altogether 6 mice bore malignant tumours, 2 of them bearing 2 each. All the 8 malignant tumours were of epithelial origin, and had penetrated the panniculus carnosus ; no metastases were seen. The experiment was terminated at the end of the 30th week.

the result that none reaches the lung in an active form. To exclude this possibility an experiment was designed in which $\beta$-propiolactone was administered intravenously via the tail vein. The local effect on the skin at the injection site, and the effect on lung tumour formation, of the intravenous injection of $\beta$-propiolactone are described in the second experimental section.

### MATERIALS AND METHODS

*Mice.*—Male and female stock albino mice of the " S " strain were used. Details of the care, feeding, and vaccination as a precaution against ectromelia, are given in a previous communication (Roe and Salaman, 1955).

*Chemical substances and their administration.*—The sources of $\beta$-propiolactone, croton oil, and acetone, and the technique of application to the skin, have been fully described elsewhere (Roe and Salaman, 1955).

Before intravenous injection mice were warmed in an incubator at 37° C. for a few minutes. Injection was made into the dorsal vein about 3 cm. from the base of the tail (proximal to the scar made by previous vaccination).

*Methods.*—The methods used for the recording of skin tumours, for the examination of mice for lung tumours at post-mortem, and for the histological examination of tissues, are fully described in a previous communication. (Roe and Salaman, 1955).

### EXPERIMENTAL

### I. *The Carcinogenic Effect of $\beta$-propiolactone on Mouse Skin*

#### (a) *The effect of $\beta$-propiolactone applied repeatedly at a subulcerative concentration (2·5 per cent)*

Ten mice were painted weekly with 0·3 ml. 2·5 per cent $\beta$-propiolactone in acetone for 52 weeks. During this period and for 3 weeks afterwards mice were examined at weekly intervals for tumours of the skin.

One mouse died after 5 weeks, the remaining 9 lived until 47 applications had been given. In these, papillomata began to appear after 27 applications, and altogether 5 mice bore papillomata. During the 40th week one papilloma on each of 2 of these 5 mice underwent a malignant change. Both tumours were removed surgically under anaesthesia, but both recurred. One metastasised to regional glands. Three weeks after the end of treatment (55th week) there were only 4 survivors, and these had to be killed because of their poor general condition.

The two malignant tumours were examined histologically ; one was a highly anaplastic carcinoma and the other a moderately well-differentiated tumour. Both had penetrated the panniculus carnosus. Fig. 1 and 2 were prepared from a section of the more differentiated tumour.

Histological examination showed that two other tumours had infiltrated the dermis but had not reached the panniculus carnosus. These were regarded as " probably " malignant (Roe, 1956).

#### (b) *The effect of early scarring, due to high concentration of $\beta$-propiolactone, on the carcinogenic effect of repeated applications of the latter*

Twenty mice were given 5 weekly applications of $\beta$-propiolactone at concentrations sufficient to produce and maintain moderate ulceration and scabbing on a majority of the mice (10, 5, 5, 10, and 5 per cent respectively). Since then, weekly applications of β-propiolactone have been continued at a sub-ulcerative concentration (2·5 per cent). During this latter treatment the ulcers and scabs caused by the earlier treatment disappeared in all mice except one. The remaining mouse was left with a curved linear scar, 2·5 cm. long, which passed diagonally across the back from the left scapula to the middle of the haunches, and remained clearly visible. Seven weeks after the beginning of treatment a papilloma appeared alongside the scar, a second papilloma appeared in a similar position after 12 weeks, and a third 3 weeks later. All three tumours, arranged in a line alongside the scar, were sessile and grew rapidly. By the 21st week all were ulcerated and obviously malignant. The mouse was killed 2 weeks later. Histological examination showed the central tumour to be an anaplastic carcinoma, and the other two to be squamous carcinomata. All had penetrated the panniculus carnosus, but no metastases were seen.

A second mouse, in which no visible scar persisted, developed a papilloma on the back during the 22nd week of the experiment. This tumour enlarged steadily, and by the 31st week appeared malignant to the naked-eye. By this time a second tumour, a papilloma, had appeared. Two weeks later the mouse died, but advanced post-mortem changes were present when its death was discovered, and no sections could be taken of the two tumours.

The experiment is at present in its 40th week, and there are 4 mice still alive. Thirteen mice have so far died tumourless.

It is concluded that β-propiolactone applied repeatedly over a period is carcinogenic for mouse skin. Not many tumours arise, but most of those that do undergo malignant transformation within a few weeks of their first appearance.

A scar caused by the application of β-propiolactone in high concentration was the site of 3 tumours, all of which appeared early and all of which became malignant.

### II. *The effects of Intravenous Injection of β-propiolactone*

### (a) *Formation of skin tumours at the site of intravenous injection of β-propiolactone*

The purpose of the following experiment was to determine whether the intravenous administration of β-propiolactone to mice would lead to the formation of lung tumours. Although the results in this respect were negative (*vide infra*), the early appearance of tumours of the skin at the site of injection on the tail was thought worthy of description.

Thirty-two mice were injected intravenously by the tail vein with β-propiolactone in sterile Ringer solution as follows :

| | |
|---|---|
| 1 mg./0·1 ml. | ( 3 ♂ mice.) |
| 3 ,, / ,, ,, | ( 3 ♂ ,, ) |
| 5 ,, / ,, ,, | (10 ♀ ,, and 3 ♂ mice.) |
| 6 ,, /0.2 ,, | ( 3 ♂ ,, ) |
| 10 ,, / ,, ,, | (10 ♀ ,, ) |

Three of the mice given 10 mg. β-propiolactone became miserable and hunched during the 24 hours after injection. Two of these recovered during the next few days, the third, and another mouse which at first appeared well, died. None of the other mice showed any signs of general intoxication. The tails of almost all the mice showed moderate or severe inflammatory changes in the neighbourhood of the site of injection. In 13 mice this inflammation proceeded to gangrene and the partial or complete loss of the tail . The records of a few of the mice which lost their tails indicated that some of the injected material had been accidently introduced outside the vein ; in the others, where no appreciable extravenous injection occurred, it is thought probable that some of the material leaked out of the vein after injection. Obvious scars persisted at the site of injection on 15 of the 17 survivors which retained their tails. Papillomata arose at the edges of these scars in 3 mice ; the first, 8 weeks after the injection of 5 mg. $\beta$-propiolactone ; the second, 9 weeks after the injection of 10 mg.; and the third, 15 weeks after the injection of 1 mg. (Fig. 3). The first of these three mice developed two further papillomata close to the scar ; these persisted for several weeks, but eventually sloughed off when the area of skin which bore them became ulcerated. Forty weeks after injection this mouse was killed ; sections taken from the ulcerated area showed chronic inflammatory changes only. The mouse which developed a papilloma at the injection site after 9 weeks developed a second similar tumour on the skin of the base of the tail overlying the vein into which the injection was made, but some distance from the point of insertion of the needle (Fig. 4). During the 48th week the first tumour in this mouse began to expand rapidly, and two weeks later the tail was amputated. Histologically the tumour, which had invaded the vertebrae, consisted of spindle cells of uncertain origin.

(b) *Failure to induce the formation of lung tumours by the intravenous injection of $\beta$-propiolactone*

As described in the previous section a total of 32 mice were given a single intravenous injection, via the tail vein, of $\beta$-propiolactone in sterile Ringer solution, in doses ranging from 1 to 10 mg.

Six months after injection 18 of the 26 survivors were killed and examined post-mortem for the presence of lung adenomata. (The remaining 8 mice are still under observation.) One mouse bore 5 lung tumours, 4 mice bore one each, and 13 mice bore none. This incidence of pulmonary adenomata is within normal limits for untreated mice of the same strain and sex.

It is concluded that single intravenous injections of $\beta$-propiolactone, in doses ranging from 1 to 10 mg., do not increase the incidence of pulmonary adenomata in " S " strain mice. On the other hand, contamination of the skin and of the subcutaneous tissues during the intravenous injection of $\beta$-propiolactone into the

---

EXPLANATION OF PLATES.

FIG. 1 and 2.—Squamous-cell carcinoma which arose on the back of a mouse after 40 weekly applications of 2·5 per cent $\beta$-propiolactone in acetone. Fig. 1 shows tumour tissue reaching down to the level of the panniculus carnosus. × 12. Fig. 2 shows infiltration by tumour cells of this muscle layer. × 270. [Staining : Haematoxylin and Eosin-Biebrich-scarlet (Salaman and Gwynn, 1951).]

FIG. 3.—Papilloma which arose 15 weeks after the intravenous injection, by the tail vein, of 1·0 mg. $\beta$-propiolactone in 0·1 ml. sterile Ringer solution.

FIG. 4.—Papillomata on the tail of a mouse following intravenous injection of 10 mg. $\beta$-propiolactone in 0·2 ml. sterile Ringer solution. The larger, more distal, tumour is situated at the site of injection ; the smaller tumour has arisen from the skin overlying the injected vein.

1

2

3

4

tail vein, or scarring following such contamination, favours the early development of papillomata at or near the site of injection.

### DISCUSSION

The results of the experiments described above are clear-cut : (i) $\beta$-propiolactone is carcinogenic for mouse-skin, (ii) scarring of the skin caused by high concentrations of $\beta$-propiolactone predisposes to the early appearance of tumours, and (iii) the intravenous administration of $\beta$-propiolactone in doses up to 10 mg. does not increase the incidence of pulmonary tumours in mice.

Walpole *et al.* (1954) described the occurrence of local sarcomata in rats following repeated subcutaneous injections of $\beta$-propiolactone. The finding that the same substance is carcinogenic for mouse-skin is therefore not entirely unexpected. Nevertheless the fact that this simple substance (molecular weight = 72) is carcinogenic for two different tissues in two distinct species is of considerable interest.

The fact that tumours arose in relation to scars caused by high concentrations of $\beta$-propiolactone may be explained in one of two ways : the five weeks of treatment at high concentrations of $\beta$-propiolactone may have been disproportionately more effective than prolonged treatment at a lower concentration, or the scarring which resulted from the high concentration may have promoted the early appearance of tumours. The close proximity of the tumours to the scars, both on the back and at the site of injection on the tail, suggest that scarring had some tumour-promoting effect. Such an effect has been the subject of many researches in the past (Pullinger, 1943, 1945*a*, 1945*b* ; Linell, 1947). Further studies of the effect of scarring on the carcinogenic action of $\beta$-propiolactone are planned.

In the experiments described above the first papilloma in a mouse painted with $\beta$-propiolactone alone appeared 7 weeks after 120 mg. had been applied. It is possible that this dose was in excess of that required to produce tumours. Nevertheless it is probable that the carcinogenicity of $\beta$-propiolactone is of a low order compared with that of 9,10-dimethyl-1, 2-benzanthracene (DMBA) ; for instance, a single application (0·3 mg.) of which has been shown to give rise to benign and malignant skin tumours (Roe, 1956). Compared with its apparently weak carcinogenic action the tumour-initiating action of $\beta$-propiolactone is strong : a single application of 7·5 mg. followed by 18 weekly applications of croton oil (0·3 ml., 0·5 per cent) gave rise to 22 papillomata* on 9 surviving mice (Roe and Salaman, 1955, p. 201). Its weakness as a carcinogen for mouse skin may be due to a deficiency in promoting power. This suggestion is supported by an experiment of our colleague Mr. R. H. Gwynn (1954, unpublished data). He applied 0·72 per cent $\beta$-propiolactone weekly, following a single initiating dose of DMBA. No tumours appeared during 20 weeks of treatment, and he concluded that $\beta$-propiolactone at this concentration does not promote tumour development. On the other hand the fact that $\beta$-propiolactone may give rise to tumours of mouse-skin by itself indicates that it is not entirely devoid of promoting activity, as appears to be the case with urethane (Salaman and Roe, 1953).

It has been suggested (Roe and Salaman, 1955) that there is a positive correlation between tumour-initiating activity for mouse skin and adenoma-inducing

* After 41 weeks, one of these papillomata showed signs of malignancy, and on microscopic examination showed penetration of the panniculus carnosus.

activity for mouse lung. The classical report of Andervont and Shimkin (1940) indicated that the intravenous route is suitable for testing substances for carcinogenic action on mouse lung, and therefore the failure to produce lung tumours in mice by the intravenous injection of β-propiolactone does not support the suggested correlation. It may be argued that failure was due to insufficient β-propiolactone reaching the lung. However this seems unlikely, since the 10 mg. intravenous doses were near the upper limit of toleration from the point of view of general toxicity.

### SUMMARY

1. Weekly applications of 2·5 per cent β-propiolactone to the backs of mice gave rise to papillomata after 27 weeks, and to epitheliomata after 40 weeks.

2. When higher concentrations (5 to 10 per cent) of β-propiolactone were given for the first 5 weeks, followed by weekly applications of 2·5 per cent, ulceration and scarring occurred. In one mouse which bore a clearly visible linear scar on the back, papillomata began to appear alongside the scar after only 7 weeks from the beginning of treatment. Three such tumours had appeared by the 15th week, and by the 21st week all three were malignant.

3. Single intravenous injections of β-propiolactone, in doses ranging from 1 mg. to 10 mg. failed to increase the incidence of pulmonary adenomata, but gave rise to severe inflammation at the site of injection, which was followed by gangrene in some mice and scar-formation in others. Papillomata arose next to the scars in 3 of the latter.

4. These findings are discussed.

We thank Dr. M. H. Salaman for his advice. The expenses of this research were partly defrayed out of a block grant from the British Empire Cancer Campaign.

### REFERENCES

Andervont, H. B. and Shimkin, M. B.—(1940) *J. nat. Cancer Inst.*, **1**, 225.
Berenblum, I. and Haran, N.—(1955) *Brit. J. Cancer*, **9**, 453.
Graffi, A., Vlamynch, E., Hoffman, F. and Schultz, I.—(1953) *Arch. Geschwulstforsch.*, **5**, 110.
Linell, F.—(1947) *Acta path. microbiol. scand.*, Suppl., **71**, 1.
Pullinger, B. D.—(1943) *J. Path. Bact.*, **55**, 301.—(1945*a*) *Ibid.*, **57**, 467.—(1945*b*) *Ibid.*, **57**, 477.
Roe, F. J. C.—(1956) *Brit. J. Cancer*, **10**, 61.
*Idem* and Salaman, M. H.—(1955) *Ibid.*, **9**, 177.
Salaman, M. H. and Gwynn, R. H.—(1951) *Ibid.*, **5**, 252.
*Idem* and Roe, F. J. C.—(1953) *Ibid.*, **7**, 472.
Walpole, A. L., Roberts, D. C., Rose, F. L., Hendry, J. A. and Homer, R. F. (1954) *Brit. J. Pharmacol.*, **9**, 306.